# Hippocampal subfields segmentation in brain MR images using generative adversarial networks

**DOI:** 10.1186/s12938-019-0623-8

**Published:** 2019-01-21

**Authors:** Yonggang Shi, Kun Cheng, Zhiwen Liu

**Affiliations:** Beijing Institute of Technology, Institute of Signal and Image Processing, School of Information and Electronics, Haidian District, Beijing, 100081 China

**Keywords:** Hippocampal subfields segmentation, Generative adversarial networks, Semantic segmentation, Fully convolution networks (FCNs), UG-net

## Abstract

**Background:**

Segmenting the hippocampal subfields accurately from brain magnetic resonance (MR) images is a challenging task in medical image analysis. Due to the small structural size and the morphological complexity of the hippocampal subfields, the traditional segmentation methods are hard to obtain the ideal segmentation result.

**Methods:**

In this paper, we proposed a hippocampal subfields segmentation method using generative adversarial networks. The proposed method can achieve the pixel-level classification of brain MR images by building an UG-net model and an adversarial model and training the two models against each other alternately. UG-net extracts local information and retains the interrelationship features between pixels. Moreover, the adversarial training implements spatial consistency among the generated class labels and smoothens the edges of class labels on segmented region.

**Results:**

The evaluation has performed on the dataset obtained from center for imaging of neurodegenerative diseases (CIND) for CA1, CA2, DG, CA3, Head, Tail, SUB, ERC and PHG in hippocampal subfields, resulting in the dice similarity coefficient (DSC) of 0.919, 0.648, 0.903, 0.673, 0.929, 0.913, 0.906, 0.884 and 0.889 respectively. For the large subfields, such as Head and CA1 of hippocampus, the DSC was increased by 3.9% and 9.03% than state-of-the-art approaches, while for the smaller subfields, such as ERC and PHG, the segmentation accuracy was significantly increased 20.93% and 16.30% respectively.

**Conclusion:**

The results show the improvement in performance of the proposed method, compared with other methods, which include approaches based on multi-atlas, hierarchical multi-atlas, dictionary learning and sparse representation and CNN. In implementation, the proposed method provides better results in hippocampal subfields segmentation.

## Background

**Fig. 1:**
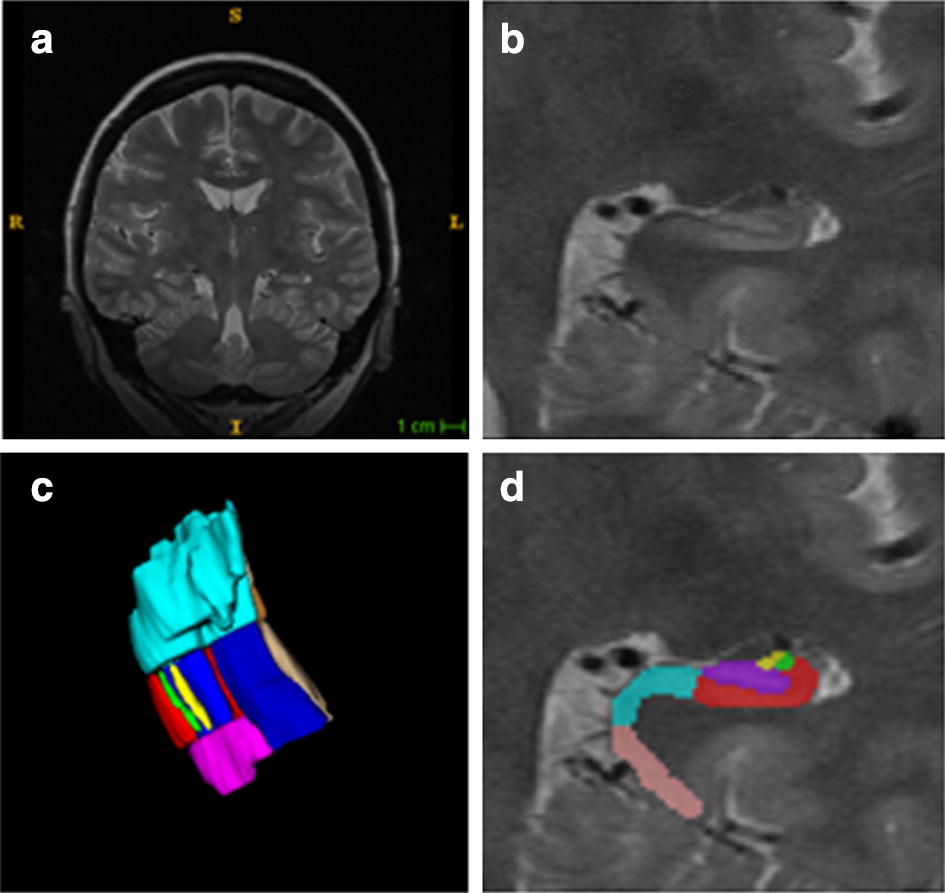
The hippocampus in T2-weighted brain MR image. **a** The T2-weighted brain image. **b** The ROI of hippocampus. **c** The 3D rendering of hippocampal. **d** The labeled image of hippocampus

The hippocampus belongs to the limbic system in the brain and plays important roles in the spatial location and long-term memory encoding and retrieval [1]. Clinically, the volume or morphology of the hippocampus and its subfields are closely related to many neurodegenerative diseases [2, 3], such as Alzheimer’s disease (AD) [4] and mild cognitive impairment (MCI) [5], it is desirable to develop automatic hippocampal subfields segmentation from brain MR image. Figure 1 shows the hippocampus in T2-weighted brain MR image.

Many promising applications on hippocampal segmentation have been reported. In 2006, Heckemann et al. [6] proposed a multi-atlas segmentation method, which registered multiple atlases to the target image and relied on label fusion to decide the label of target voxel. There was a main disadvantage of the multi-atlas method, which the segmentation result was highly dependent on the registration algorithms. Patch-based methods [7, 8] based on multi-atlas segmentations, which identified local similarities between the atlases and the target image at certain patch level, effectively eliminated the registration errors caused by the registration between the atlases and target image. Nevertheless, atlas patches with high similarity might still matched to the wrong labels because image similarities over small image patch may lead to the local optima. To avoid being trapped into the local optima, sparse coding methods [9, 10] were proposed to ensure that several highly relevant atlas patches are selected to represent the target patch. Discriminative dictionary learning [11, 12] was utilized to guide the representation transition from similarity to label. However, methods based on sparse coding and discriminative dictionary learning still have the disadvantage in selecting the best patches to reconstruct the target patch due to the limitation in discriminative ability of the model.

In recent years, deep learning models have achieved impressive state-of-the-art performance over traditional machine learning approaches on a variety of computer vision tasks, including image segmentation, classification, and recognition. Alex et al. [13] proposed Alex-net, which won the ImageNet large scale visual recognition challenge (ILSVRC) in 2012, setting off an application upsurge of deep learning in the field of computer vision and image processing. Ciprian et al. [14] utilized the improved VGGNet to achieve the classification of MRI images in the hippocampus of AD, MCI, and normal brain regions. The accuracy was greatly improved compared with the traditional methods. Long et al. [15] built fully convolutional networks (FCNs), which is one kind of end-to-end deep network, to achieve the pixel-wise classification of input images. Nevertheless, FCNs discard valuable context information because the methods consider one single pixel in the images as conditionally independent from the others. The U-net architecture [16] with very few training images, an extension of FCNs, produced fast and precise segmentation of images by equipping with a large number of feature channels, which allow the modified architecture to propagate context information to subsequent layers.

Deep neural networks have some intrinsic shortcomings, such as the neglect of the interrelationship between pixels in the image, and the assumption that training data and testing data follow the same distribution. In order to overcome these shortcomings, Goodfellow et al. [17] proposed generative adversarial network**s** (GAN) constituting a novel architecture for estimating generative model through an adversarial process. Several methods [18–21] were proposed to solve the problem that the GAN network structure is unstable and difficult to train. Luc et al. [22] applied GAN to the natural image segmentation for the first time, achieving higher accuracy segmentation results by training the VGG16-based generative adversarial network.

In this paper, we propose a generative adversarial network model with the modified U-net (named UG-net) and the adversarial model to achieve an end-to-end, high-accuracy, fully automated segmentation of the hippocampal subfields of brain MRI images. In this method, some steps like candidate preselection or complex post-processing are abandoned. The experiments are performed on the ADNI dataset to validate the proposed method. The experimental results show that the proposed method outperforms the state-of-the-art method**s**.

## Methods

**Fig. 2:**
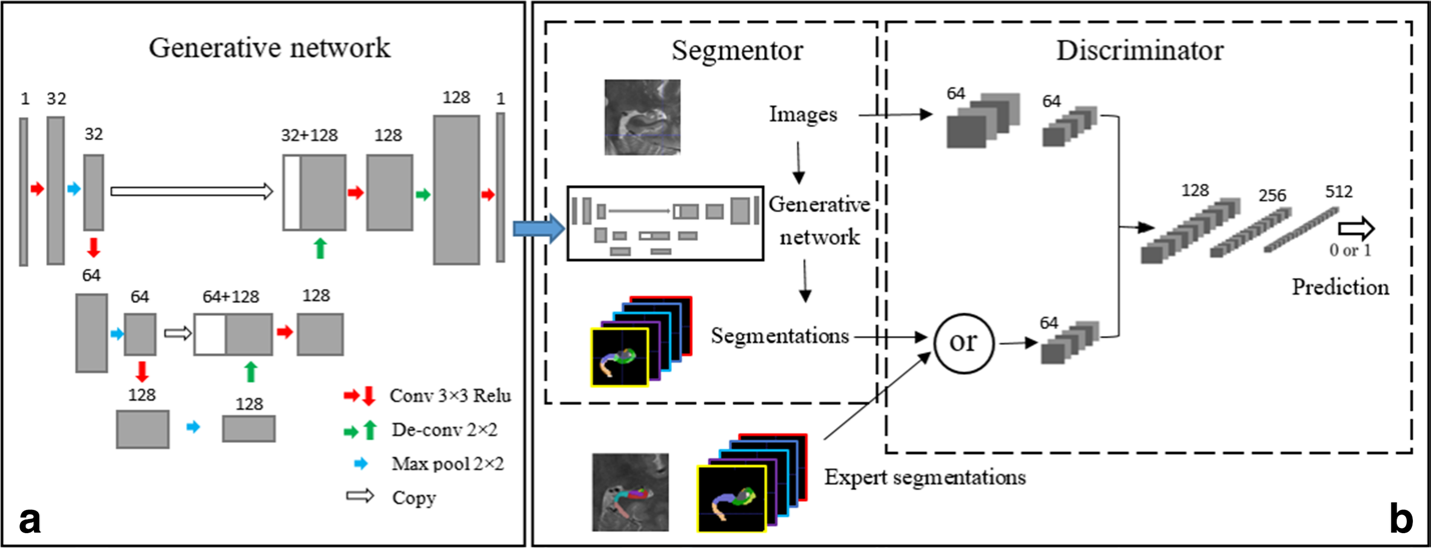
Illustration of the proposed GAN networks. **a** The generative network named UG-net, which is the modification of U-net, taking brain MR images as input and producing per-pixel class predictions. **b** The overview of GAN network including the generative model and the adversarial model

In this section, we present the general framework for adversarial training of our hippocampal subfields segmentation models. Figure 2 describes the proposed model in details. The model consists of two major parts. The first part is generative network based on the modified U-net, which is trained to conduct the 2D segmentation of the slices extracted from brain nuclear magnetic image. The other part, an adversarial network with convolutional neural network is employed to discriminate the expert annotation and the segmentation images generated by generative network.

### Generative adversarial network

The generative adversarial network, which consists of two components: a generator G and a discriminator D, is a deep learning framework that trains the generative model and the discriminative model alternately. The general idea of the generative adversarial network is an adversarial process amongst the models pitting against each other to improve the performance of the networks, where the generator counterfeits the sample images to deceive the discriminator and the discriminative model determines whether the images are fake or not. The sufficient competition and confrontation between the models leads to the improved performance of the models so that the generative images are indistinguishable from the real sample images.

The formula of the generative adversarial network optimal training process is as follows:

1 $$ V\left( {G,D} \right) = \mathop {\hbox{min} }\limits_{G} \mathop {\hbox{max} }\limits_{D} \left( {{\rm E}_{{x\sim P_{data} \left( x \right)}} \left[ {\log D\left( x \right)} \right] + {\rm E}_{{z\sim P_{z} \left( z \right)}} \left[ {\log \left( {1 - D\left( {G\left( z \right)} \right)} \right)} \right]} \right) $$

where *x* is a data sample. GANs have achieved state-of-the-art in some generation tasks, such as image synthesis, video generation. The conditional GAN has been proposed to solve some ill-posed problems, such as text-to-image translation, image-to-image translation, and image super-resolution. The conditional GAN receives an additional input as a condition to guide the generation of the images.

As shown in Eq. (2), the combination loss function that consists of two terms is used to optimize the models. The first term is a multi-class cross-entropy term encouraging the generative model to generate the segmentations of high accuracy. We use *g*(*x*) to denote the class probability map over *M* classes with the size of *H *× *W *× *M* produced by the generative model given an input image *x* with the size of *H *× *W*. The second term, which is based on the adversarial model, will be large if the adversarial model can discriminate the generative segmentations from the expert manual segmentations. This term will penalize the mismatches in the higher-order label statistics due to the fact that the adversarial model has a field-of-view covering the entire image. We use *a*(*x*, *y*)∈[0,1] to denote the probability predicted by the adversarial model that *y* is the expert manual segmentation label map of *x* or the label map produced by the generative model *g*(·). For a dataset of *N* training images *x*_*n*_ with the corresponding label maps *y*_*n*_, the loss function is defined as follows

2 $$ \ell \left( {\theta_{g} ,\theta_{a} } \right) = \sum\limits_{n = 1}^{N} {\ell_{mce} \left( {g\left( {x_{n} } \right),y_{n} } \right)} - \left[ {\ell_{bce} \left( {a\left( {x_{n} ,y_{n} } \right),1} \right) + \ell_{bce} \left( {a\left( {x_{n} ,g\left( {x_{n} } \right)} \right),0} \right)} \right] $$

where *θ*_*g*_, *θ*_*a*_ are the parameters of the generative model and of the adversarial model respectively. In the Eq. (2), $\ell_{mce} \left( {\hat{y},y} \right) = - \sum\nolimits_{i = 1}^{H \times W} {\sum\nolimits_{m = 1}^{M} {y_{im} } } \ln \hat{y}_{im}$ denotes the multi-class cross-entropy loss of the predictions $\hat{y}$, and $\ell_{bce} \left( {\hat{a}a} \right){ = } - \left[ {a\ln \hat{a} + \left( {1 - a} \right)\ln \left( {1 - \hat{a}} \right)} \right]$ denotes the binary cross-entropy loss. When training the generative model, we minimize the loss with respect to *θ*_*g*_, while maximizing it with respect to *θ*_*a*_ when training the adversarial model.

### The generative model

Based on the U-net, the generative model named UG-net, utilizes the encoder-decoder architecture as shown in Fig. 2. The encoder, consists of several conventional “convolution + pooling” layers, attempts to extract the high-level features as opposed to the decoding part employed to reconstruct the segmentation ground truth label maps by upsampling layers. Highly condensed features, which are very effective for image segmentation, are extracted by the convolution layers, but some important local information has been missed in this processing. To retain the local information, the feature maps extracted by the convolution layers are concatenated with the corresponding output feature maps of the upsampling layers. Some modifications are made for the original U-net in the architecture and training strategies to suit our task and dataset. In the paper, instead of using two consecutive convolution layers prior to the pooling layer in U-net, we remove one of the convolution layers to reduce the parameters of the model. This modification aims to prevent overfitting since U-net may be easily overfitted in our task with more parameters. In addition, zero padding convolution layers are adopted to maintain the spatial dimension of the images and feature maps through each convolution layer. Data augmentation and dropout operations are also used to against the overfitting on several upsampling layers. In the last convolution layers, we only use one 3 × 3 filter to reduce the number of output feature channels to one, same to the channels of the expert segmentations.

### The adversarial model

**Fig. 3:**
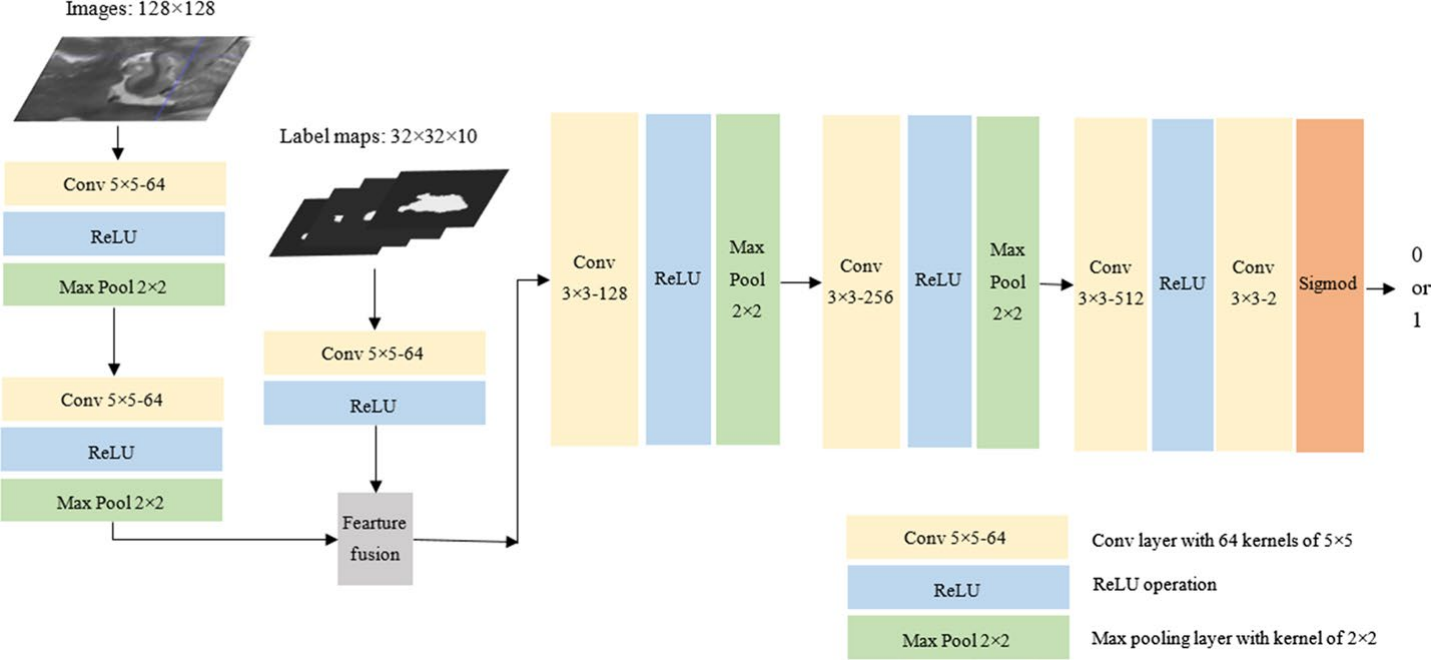
The details of adversarial model architecture in our experiments

The adversarial model architecture is illustrated in Fig. 3. The model takes the hippocampal image and the corresponding label map as input. The label map is either the expert segmentations or produced by the generative model. At the beginning, the images and the label maps are processed by two separate branches to allow different level representations for the two different signals. Following the observation of Pinherio et al. [23], the same number of channels is set up for each input signal to avoid that one of them dominates the other when fed to the subsequent layers. The two inputs are represented with 64 channels by concatenating the two signal branches. Then the signals are delivered into a series of convolution and max-pooling layers, thus a binary class probability is produced to determine whether the label map is the expert segmentation or not.

## Experiments

### Dataset

For validation of the proposed model, a dataset of brain MR images obtained from the CIND Center in San Francisco, USA was used. The dataset contains 3D T1-weighted and T2-weighted brain MR images of 32 subjects, aged between 38 and 82 years old, with 18 males and 14 females. Among them, there were 21 normal persons, 4 MCI patients, and 7 AD patients. The resolution of T1-weighted image and T2-weighted image is 1 × 1×1 mm^3^ and 0.4 × 0.5 × 2 mm^3^, respectively. The expert segmentations with high labeling credibility, which can be regarded as ground truth, were labeled manually by several clinically experienced physicians and experts. First, the brain MR images were registered using the linear registration method based on the affine transformation model in insight toolkit (ITK) library and the symmetric diffeomorphic registration method based on the mutual information [24]. Then, the registered images were normalized to the range [0, 255] using the linear transformation method [25]. Then, Convert 3D, an image processing tool, was employed to extract the region of interest from brain MR images. The size of the region of interest was 112 × 103 × 24. Since the proposed model takes 2D planar images as input, twenty-four 2D image slices were extracted from each 3D MR image as sample images. There are 768 different images before the data augmentation. In order to prevent overfitting, data augmentation methods such as rotation, translation, and adding Gaussian noise were performed on the slices to increase the amount of data [13, 26], after which the total number of sample images is 5500. The MRI images of the dataset are available at http://isip.bit.edu.cn/kyxz/xzlw/134492.htm.

### Optimization function

The generative model and the adversarial model were trained alternately in the experiments. First, we minimize the multi-class cross-entropy loss to train the generative model, which encourages the generative model to produce the accurate segmentations that are hard to distinguish from the expert segmentations by the adversarial model. The terms relevant to generative model in the loss function (2) are

3 $$ \ell \left( {\theta_{g} } \right) = \sum\limits_{n = 1}^{N} {\ell_{mce} \left( {g\left( {x_{n} } \right),y_{n} } \right)} - \ell_{bce} \left( {a\left( {x_{n} ,g\left( {x_{n} } \right)} \right),0} \right) $$

In training step, $- \ell_{bce} \left( {a\left( {x_{n} ,g\left( {x_{n} } \right)} \right),0} \right)$ was replaced with ${ + }\ell_{bce} \left( {a\left( {x_{n} ,g\left( {x_{n} } \right)} \right),1} \right)$ [17]. It can be understood that we maximize the probability that the adversarial model predicts $g\left( {x_{n} } \right)$ to be the expert segmentations instead of minimize the probability to be generative segmentations. The reason of the modification is that it leads to a stronger gradient descent when the segmentation model is trained. It is proven that this modified update is indeed of great significance to speedup the training process.

When the adversarial model is trained, it is obvious that only the second term in the loss function (2) depends on the adversarial model, so we can minimize the following binary classification loss to train the adversarial model.

4 $$ \ell \left( {\theta_{a} } \right){ = }\ell_{bce} \left( {a\left( {x_{n} ,y_{n} } \right),1} \right) + \ell_{bce} \left( {a\left( {x_{n} ,g\left( {x_{n} } \right)} \right),0} \right) $$

### Training and testing

The models are trained on a Nvidia GPU. 600 image slices of 5 subjects were chosen for testing from the total dataset, and 4900 slices of the other 27 subjects were used to train the proposed model. The training and testing strategies have been same for all evaluated schemes. The training processing is performed using tenfold cross validation. In each cross-validation permutation, one subsample is left as the validation data for testing the model, and the remaining 9 subsamples are used as training data. The generative model was trained for 100,000 iterations, with the batch size of 5, using an initial learning rate of 10^−5^, which is halved every 5000 iterations. For parameter optimizations the stochastic gradient descent (SGD) with momentum was used. The momentum was set as 0.99 and weight decay as 10^−4^. During the training stage, the adversarial model was trained using fixed learning rate of 10^−5^ on 3 batches while the generative model was trained on one batch.

## Results

In this section, we evaluate the proposed GAN method by using the MRI images of several subjects, which are different from the subjects for training the model. We randomly choose 5 subjects containing 600 image slices from the dataset to validate the methods, and the other 27 subjects comprising 4900 slices are used to train the model. The experiment was performed by using UG-net (only the generative model without adversarial training) and our method with GAN (the whole generative adversarial network) respectively. In addition, to verify the performance of the proposed model, the segmentation results of UG-net and GAN network are analyzed by the cross-validation experiment. Moreover, the proposed method results are compared with some state of the art work [8, 10, 27, 28] that focus on hippocampus segmentation.

The Dice similarity coefficient (DSC) is used as a statistical validation metric to evaluate the accuracy of segmentation of MR images. It is defined as follows:

5 $$ DSC\,(S_{1} ,\,S_{2} ) = \frac{{ 2\times \left| {S_{1} \cap S_{2} } \right|}}{{\left| {S_{1} } \right| + \left| {S_{2} } \right|}} $$

where *S*_*1*_ represents the generative segmentations by the two networks and *S*_*2*_ represents the expert segmentations respectively. Meanwhile, $\left| {\cdot} \right|$ indicates the number of pixels in the area. It is important to note that the segmentation is performed on the 2D slices, but the hippocampus segmentation accuracy was calculated at the volume level.

**Fig. 4:**
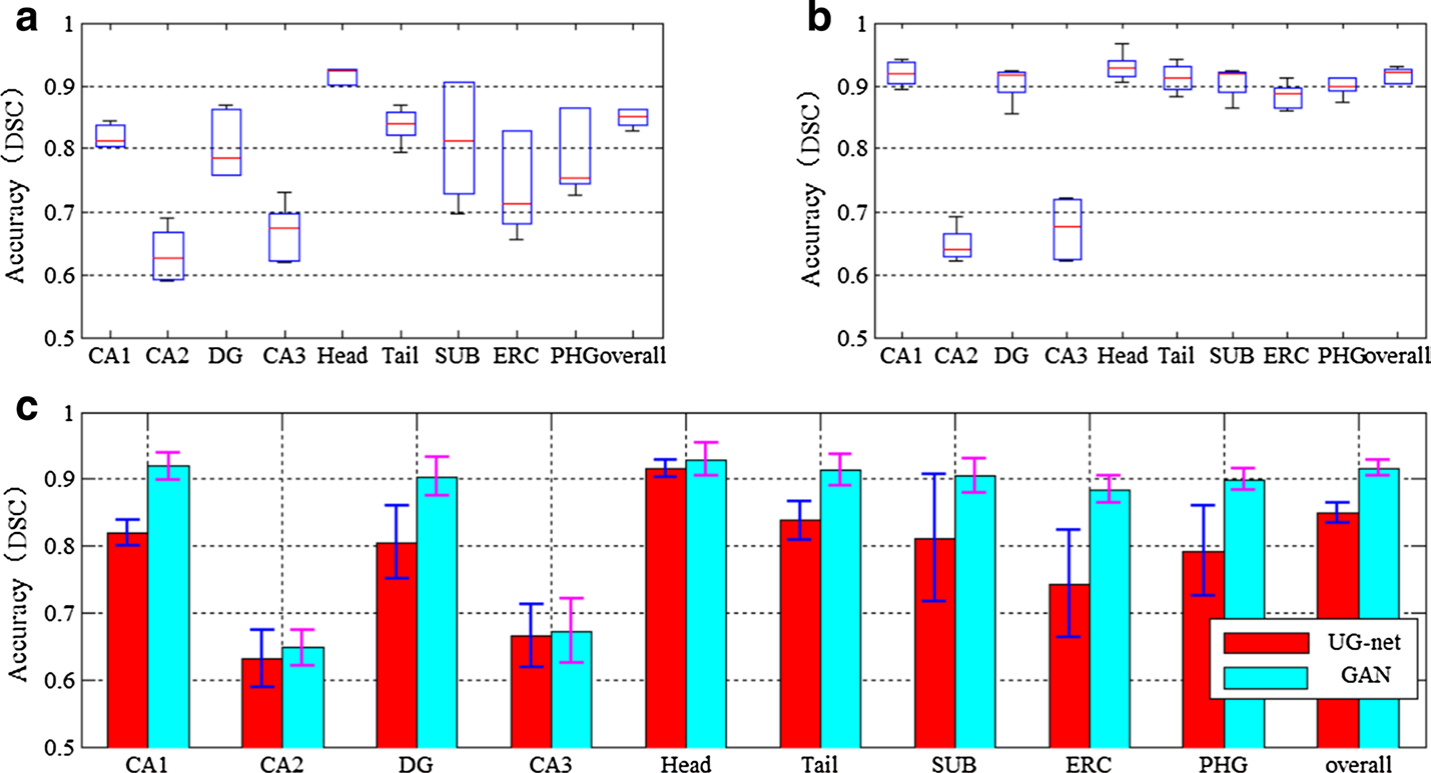
Comparison between the mean and standard deviation of segmentation accuracies of UG-net and GAN. **a** The segmentation accuracy of UG-net. **b** The segmentation accuracy of GAN. **c** The comparison of segmentation accuracies between UG-net and GAN

**Fig. 5:**
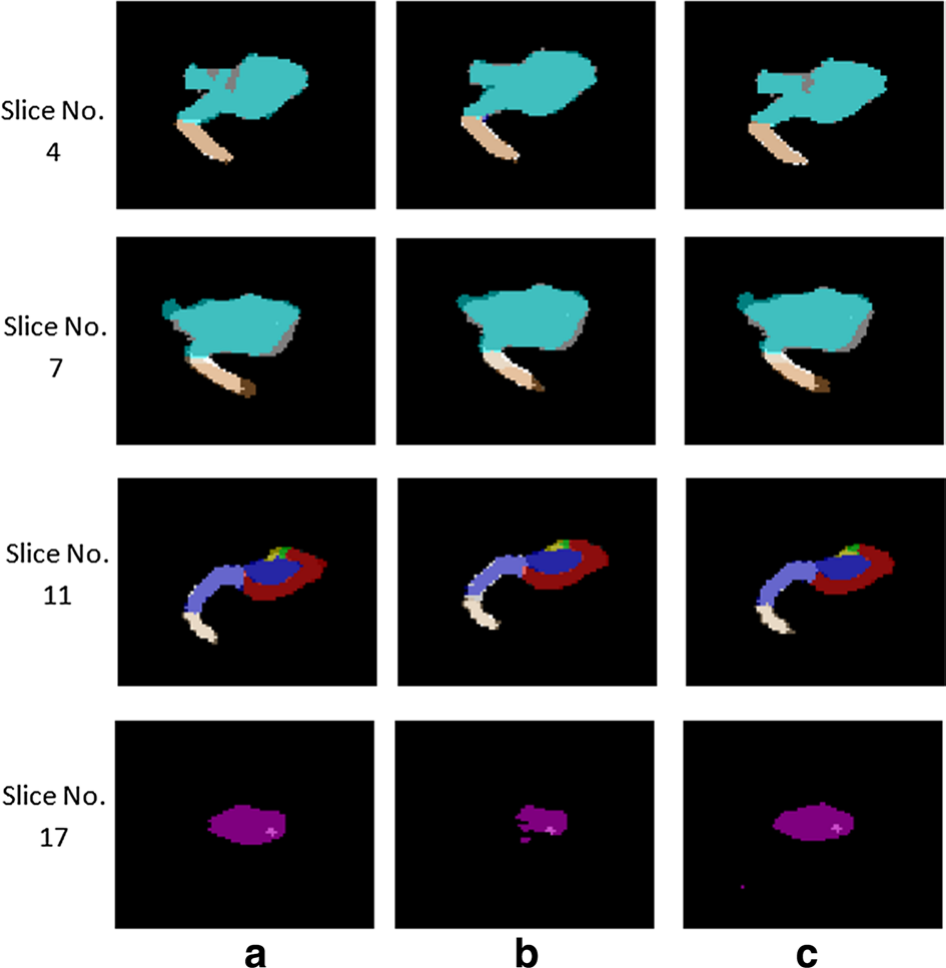
The different methods segmentation results and the expert segmentation in some slices of brain MR image. **a** The segmentation of expert. **b** The segmentation of UG-net. **c** The segmentation of GAN

Figure 4 provides the mean and standard deviation of segmentation accuracy for hippocampus segmentation results of UG-net (without adversarial training) and the proposed method (with adversarial training) from fivefolds. Moreover, the different methods segmentation results and the expert segmentation in various slices of brain MR image are given in Fig. 5. The results in the figures show that UG-net and the proposed method achieve high segmentation accuracy. For segmentation of larger subfield such as head of hippocampus, the accuracy reaches 91.5% and 92.9%, respectively. For segmentation of smaller subfields, such as CA2 and CA3, the accuracy is also relatively high. The overall mean accuracy of segmentation of all hippocampal subfields has reached 84.9% and 91.6%, respectively.

**Fig. 6:**
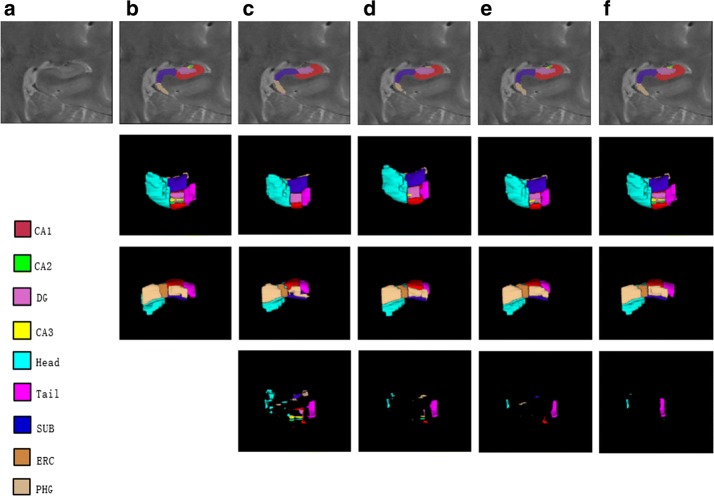
Comparison between the different methods segmentation results and the expert segmentation. **a** The ROI of hippocampus in brain MR image. **b** The segmentation of expert. **c** The segmentation of Sparse Coding and Dictionary Learning. **d** The segmentation of CNN. **e** The segmentation of UG-net. **f** The segmentation of our method

Figure 6 delivers the results comparison of the proposed method, some other methods and expert segmentation. The compared segmentation methods include dictionary learning and sparse representation, CNN-based method, UG-net and our proposed method. As shown in Fig. 6, the first row shows the segmentation of hippocampal subfields in coronal plane by different segmentation methods. The second and third rows are the three-dimensional rendering of hippocampal subfields. And the fourth row is the three-dimensional rendering of segmentation results difference between different methods and the expert manual delineation. Figure 6 shows that the segmented hippocampus three-dimensional rendering using the proposed method are much closer to the expert segmentation than the other methods.

Besides, we compare the proposed model with some state of the art methods that focus on hippocampus segmentation. Among the methods, multi-atlas approach and hierarchical multi-atlas approach focus on the whole hippocampus segmentation, while methods based on dictionary learning and sparse representation and CNN focus on the hippocampal subfields segmentation. Please note that the results are compared on the same dataset with dictionary learning and sparse representation and CNN methods, and the different dataset with multi-atlas and hierarchical multi-atlas approaches. We do not intend to make a head-to-head quantitative comparison with multi-atlas and hierarchical multi-atlas approaches as we focus on the segmentation of the hippocampus subfields rather than the whole hippocampus (multi-atlas and hierarchical multi-atlas approaches do). On the other hand, we just qualitatively compared the proposed method with multi-atlas and hierarchical multi-atlas approaches to show that our method has the competitive performance in the whole hippocampus segmentation. Nevertheless, both the highest segmentation accuracy of overall and that of subfields are achieved by our proposed methods, as shown in Table 1. For whole hippocampal segmentation, the DSC is increased by 2.63% than multi-atlas approach. For the subfields, especially for ERC and PHG, the segmentation accuracy is also significantly increased 20.93% and 16.30% respectively than the method based on CNN. For both cases, it shows that the proposed method with GAN has achieved the improved performance in segmenting no matter whole hippocampus or hippocampal subfields.

**Table 1:** Quantitative evaluation of different segmentation methods (DSC)

Methods	Hippocampal subfields									Overall
	CA1	CA2	DG	CA3	Head	Tail	SUB	ERC	PHG	
Multi-Atlas [8]	–	–	–	–	–	–	–	–	–	0.8925
Hierarchical Multi-Atlas [10]	–	–	–	–	–	–	–	–	–	0.8850
Dictionary learning and sparse representation [27]	0.804	0.538	0.807	0.548	0.890	0.772	0.751	0.743	0.620	–
CNN [28]	0.836	0.603	0.843	0.641	0.894	0.865	0.810	0.731	0.773	–
UG-net	0.820	0.631	0.805	0.666	0.915	0.838	0.811	0.743	0.792	0.849
Our method	0.919	0.648	0.903	0.673	0.929	0.913	0.906	0.884	0.899	0.916

## Discussion

It is well known that traditional convolutional neural networks can automatically extract high-dimensional image features. However, some local information is missed after a series of convolution and pooling operations in the networks, and the local information is very important for accurate segmentation of small targets. Therefore, this paper employs UG-net to preserve valuable local information through the skip-connection, which achieves accurate segmentation of the smaller hippocampus subfields. As shown in Figs. 5 and 6, the proposed model implements improved performance on small regions, such as ERC and PHG, which verifies that UG-net can retain more local information.

Different from the traditional convolutional neural network, the generative adversarial network attempts to fit the distribution of the generated segmentation results to the one of real samples. The adversarial training can maintain the interrelation between the pixels and improve the spatial consistency among the class labels. As shown in Figs. 4 and 5, generally, the proposed model has achieved higher segmentation accuracy than UG-net both in hippocampal subfields and in overall hippocampus. Moreover, the adversarial training smoothens and strengthens the edges of class labels on large regions, e.g. the first row in Fig. 6. These verify the hypothesis that adversarial training can overcome the intrinsic shortcomings of common deep neural networks, such as missing the interrelationship between pixels and assuming that training data and test data follow the same distribution. The figures show that the adversarial training strategy efficiently reduces overfitting, generates a regularization effect, and outperforms UG-net in accuracy of subfields segmentation. It is not hard to find that the accuracy standard deviation of the proposed model with GAN is higher than the one of UG-net.

Although the proposed model has achieved higher accuracy in the segmentation of the hippocampal and the larger subfields, it doesn’t perform well enough in the smaller hippocampal subfields and there is still some work to improve the performance. The reason is that the subfields are so small that the sample voxels are too few, which affects the segmentation results of the model. Furthermore, since the registration methods in preprocessing may impact the image segmentation results, further research is needed to evaluate if different registration methods have any effect on the performance of proposed method.

## Conclusion

In this paper, a method based on generative adversarial networks was proposed to achieve the higher accurate hippocampal subfields segmentation. By building an UG-net model and an adversarial model and training the model against each other alternately, we achieved superior performance in hippocampal subfields segmentation on the dataset of CIND. Compared with the existing methods, the proposed model has the following advantages: (1) the GAN networks retain spatial consistency among the generated class labels and smoothen the edges of class labels on segmented region. (2) UG-net can extract more local information and improve the accuracy of the segmentation of small subfields with retaining the interrelationship between pixels. (3) In terms of the intrinsic quality of classification, the proposed model tends to fit the distribution of the generated segmentation results to the one of real samples. Exploring a more efficient training strategy or model structure is our next step. And we will also attempt to apply the proposed method to the segmentation of other physiological tissues such as kidney cortex and brain tumors.

## References

[1] Lim HK, Hong SC, Jung WS, Ahn KJ, Won WY, Hahn C, Kim I, Lee CU (2012). Automated hippocampal subfields segmentation in late life depression. J Affect Disord.

[2] Voets NL, Bernhardt BC, Kim H, Yoon U, Bernasconi N (2010). Increased temporolimbic cortical folding complexity in temporal lobe epilepsy. Neurology..

[3] Kim H, Mansi T, Bernasconi N, Bernasconi A (2012). Surface-based multi-template automated hippocampal segmentation: application to temporal lobe epilepsy. Med Image Anal.

[4] Hobbs KH, Zhang P, Shi B, Smith CD. Quad-mesh based radial distance biomarkers for Alzheimer’s disease. In: 2016 IEEE 13th international symposium on biomedical imaging (ISBI). 2016. p. 19–23. 10.1109/isbi.2016.7493201.

[5] Nestor SM, Gibson E, Gao FQ, Kiss A, Black SE (2013). A direct morphometric comparison of five labelling protocols for multi-atlas driven automatic segmentation of hippocampus in Alzheimer’s disease. NeuroImage..

[6] Heckemann RA, Hajnal JV, Aljabar P, Rueckert D, Hammers A (2006). Automatic anatomical brain MRI segmentation combining label propagation and decision fusion. NeuroImage..

[7] Coupé P, Manjón JV, Fonov V, Pruessner J, Robles M, Collins DL (2011). Patch-based segmentation using expert priors: application to hippocampus and ventricle segmentation. NeuroImage..

[8] Wang H, Suh JW, Das SR, Pluta JB, Craige C, Yushkevich PA (2013). Mult-atlas segmentation with joint label fusion. IEEE T Pattern Anal..

[9] Wu GR, Wang Q, Zhang D, Nie F, Huang H, Shen DG (2014). A generative probability model of joint label fusion for multi-atlas based brain segmentation. Med Image Anal.

[10] Wu GR, Kim M, Sanroma G, Wang Q, Munsell BC, Shen DG (2015). Hierarchical multi-atlas label fusion with multi-scal feature representation and label-specific patch partition. NeuroImage..

[11] Tong T, Wolz R, Coupé P, Hajnal JV, Rueckert D (2013). Segmentation of mr images via discriminative dictionary learning and sparse coding: application to hippocampus labeling. NeuroImage..

[12] Deng Y, Rangarajan A, Vemuri BC. Supervised learning for brain mr segmentation via fusion of partially labeled multiple atlases. In: 2016 IEEE 13th international symposium on biomedical imaging (ISBI). 2016. p. 633–7. 10.1109/isbi.2016.7493347.

[13] Krizhevsky A, Sutskever I, Hinton GE (2012). ImageNet classification with deep convolutional neural networks. Int Conf Neural Inform Process Syst.

[14] Ciprian DB, Olivia JD, David EH, Naval P. DemNet: a convolutional neural network for the detection of Azheimer’s disease and mild cognitive impairment. IEEE Region 10 Conference (TENCON). 2016; p.3724–7. 10.1109/tencon.2016.7848755.

[15] Shelhamer E, Long J, Darrell T (2017). Fully convolutional networks for semantic segmentation. IEEE Trans Pattern Anal Mach Intell.

[16] Ronneberger O, Fischer P, Brox T. U-net: Convolutional networks for biomedical image segmentation. Int Conf MICCAI. 2015; p. 234–41. 10.1007/978-3-319-24574-4_28.

[17] Goodfellow I, Pouget-Abadie J, Mirza M, Xu B, Warde-Farley D, Ozair S, Courville A, Bengio Y. Generative adversarial nets. Proc Adv Neural Inf Process Syst. (NIPS) 2014; p. 2672–80.

[18] Mirza M, Osindero S, Conditional generative adversarial nets. Comput Sci. 2014; p. 2672–80.

[19] Chen X, Duan Y, Houthooft R, Schulman J, Sutskever, Abbeel P. InfoGAN: interpretable representation learning by information maximizing generative adversarial nets. In: Proc Adv Neural Inf Process Syst. (NIPS), 2016.

[20] Arjovsky M, Chintala S, Bottou L. Wasserstein generative adversarial networks. In: Proceedings of the 34th international conference on machine learning (ICML). 2017; 70: 214–23.

[21] Che T, Li Y, Jacob AP, Bengio Y, Li W. Mode regularized generative adversarial networks. In: International conference on learning representations (ICLR), 2017.

[22] Luc P, Couprie C, Chintala S, Verbeek J. Semantic segmentation using adversarial networks. In: NIPS workshop on adversarial training, 2016.

[23] Pinheiro PO, Lin T, Collobert R,. Dollár P. Learning to refine object segments. In: Proc Eur Conf Comput Vis. (ECCV). 2016; p. 75–91. 10.1007/978-3-319-46448-0_5.

[24] Yushkevich PA, Wang HJ, Das SR, Craige C, Avants BB, Weiner MW, Mueller S (2010). Nearly automatic segmentation of hippocampal subfields in vivo focal T2-weighted MRI. NeuroImage..

[25] Nyúl LG, Udupa JK (1999). On standarding the MR image intensity scales. Magnet Reson Med..

[26] Pereira S, Pinto A, Alves V, Silva CA (2016). Brain tumor segmentation using convolutional neural networks in MRI images. IEEE Trans Med Imaging..

[27] Shi YG, Wang DQ, Liu ZW (2015). Segmentation of hippocampal subfields using dictionary learning and sparse representation. J Image Graph..

[28] Shi YG, Hao HY, Liu ZW (2018). Cascaded convolutional neural network based hippocampal subfields segmentation. J Image Graph..

